# Anesthetic Management and Perioperative Predictors of Outcomes in Combined Body Contouring Procedures

**DOI:** 10.1007/s00266-026-05991-0

**Published:** 2026-06-15

**Authors:** Taylan Sahin, Ali Sait Kavakli, Yasemin Aydinli, Nese Kutluturk Sahin, Alp Hepsev

**Affiliations:** 1 Department of Anesthesiology and Reanimation, Faculty of Medicine, Istinye University, Istanbul, Turkey; 2 Department of Plastic Surgery, Faculty of Medicine, Istinye University, Istanbul, Turkey; 3 Department of Radiology, Istanbul Physiotherapy and Rehabilitation Training and Research Hospital, Istanbul, Turkey; 4 Department of Anesthesiology and Reanimation, Faculty of Health Sciences, Istanbul Gelisim University, Istanbul, Turkey

**Keywords:** Combined body contouring, Anesthetic management, Perioperative predictors, ERAS protocol, Postoperative complications, Patient safety

## Abstract

**Background:**

Brazilian Butt Lift (BBL), liposuction, and abdominoplasty are increasingly being performed in combination to enhance esthetic results and reduce recovery time. However, this approach may lead to prolonged surgical times, complex anesthesia management, and an increased risk of potential complications. This study aims to evaluate the relationship between intraoperative variables and postoperative complications in patients undergoing combined body contouring surgery.

**Methods:**

A total of 1120 patients who underwent esthetic surgery between 2020 and 2025 were included in this retrospective cohort study. Patients were divided into two groups: Group 1 (*n* = 550) underwent BBL, liposuction, and abdominoplasty; Group 2 (*n* = 570) underwent only BBL and liposuction. Demographic data, intraoperative parameters, laboratory values, and postoperative complications were analyzed. Multivariate logistic regression and ROC analyses were performed to identify factors predicting complications.

**Results:**

In Group 1, the duration of surgery, amount of crystalloid fluid administered, volume of aspirate removed by liposuction, amount of fat injected, and urine output were significantly higher (*p* < 0.001). The total complication rate was 0.8%, with postoperative complications developing in only 9 patients. Three of these patients (33.3%) had a history of bariatric surgery. In the logistic regression analysis, prolonged surgical duration (OR = 2.12) and a history of surgery (OR = 1.87) were associated with the development of complications; however, neither reached statistical significance (*p* > 0.05). In the ROC analysis (multivariate), the model’s discriminatory power was high (AUC = 0.94).

**Conclusions:**

Combined esthetic surgeries appear to be safe when performed by an experienced surgical–anesthesia team with close intraoperative monitoring. Surgical duration and previous surgical history may be associated with complications; however, larger prospective studies are needed to confirm these findings.

**Level of Evidence III:**

This journal requires that authors assign a level of evidence to each article. For a full description of these Evidence-Based Medicine ratings, please refer to the Table of Contents or the online Instructions to Authors www.springer.com/00266.

## Introduction

In recent years, body contouring surgeries have shown a significant increase in esthetic surgical practice. Procedures such as the Brazilian Butt Lift (BBL), abdominoplasty, and 360-degree liposuction are frequently preferred by both patients and surgeons to achieve more ideal body contours [1]. Although these surgeries have the potential to increase esthetic satisfaction, they require careful clinical planning and management due to their technical difficulties and physiological demands.

Performing multiple esthetic procedures in the same session results in longer surgical times and a more complex anesthesia process. The literature indicates that combined plastic surgeries may have higher complication rates in the postoperative period [2, 3]. Among these complications are conditions such as pulmonary embolism, hematoma, atelectasis, and pulmonary edema, which can potentially lead to serious consequences [4]. However, publications show that factors such as the surgical team’s experience, anesthesia protocols, fluid management, and intraoperative monitoring can reduce these risks [5].

Anesthesia management is a critical component that directly affects success in combined and high-volume esthetic surgeries. Maintaining hemodynamic stability, intraoperative fluid balance, body temperature, and monitoring tissue perfusion during prolonged operations are decisive in preventing surgical complications [6]. The use of invasive monitoring techniques (e.g., arterial blood pressure monitoring) optimizes intraoperative decision-making while also facilitating early diagnosis and treatment of postoperative complications [7]. The literature indicates that working with a fixed anesthesia team and standardizing protocols has been shown in various studies to increase patient safety by reducing complication rates [8]. In this context, the quality of anesthesia practices and the intraoperative monitoring process are as important factors for the success of esthetic surgery as the surgical technique itself.

Determining safe practice conditions for combined esthetic surgeries and identifying factors contributing to the risk of complications are important requirements for both patient safety and surgical planning. Therefore, clinical evaluations in this field are valuable for guiding future practices.

In this context, the aim of this study is to evaluate the safety of combined and high-volume procedures frequently performed in esthetic surgery practice, to determine complication rates, and to analyze perioperative and intraoperative risk factors associated with these complications.

## Methods

### Study Design and Patient Selection

This retrospective cohort study was conducted at a single-center tertiary healthcare institution and included patients who underwent esthetic body contouring surgery between January 2020 and March 2025. Approval for the study was obtained from the Istinye University Ethics Committee (2025/05). Patients were divided into two groups based on the type and scope of the surgical procedure performed. Group 1 consisted of patients who underwent combined procedures, including Brazilian Butt Lift (BBL), 360-degree liposuction, and abdominoplasty (*n* = 550); Group 2 consisted of patients who underwent only BBL and 360-degree liposuction, without abdominoplasty (*n* = 570).

Inclusion criteria were age 18-60 years, ASA physical status I–II, complete perioperative data, and elective surgery. Patients with incomplete medical records, pre-existing cardiac or renal dysfunction, and those undergoing emergency or revision surgery were excluded.

### Anesthesia and Intraoperative Management

A standard general anesthesia protocol was applied to all patients. Anesthesia induction was performed intravenously with propofol (2–2.5 mg/kg), fentanyl (1–2 mcg/kg), and rocuronium (0.6 mg/kg). After intubation, anesthesia maintenance was achieved with sevoflurane (1–2%) in an oxygen/air mixture (FiO_2_: 0.5). Additionally, continuous remifentanil infusion (0.1–0.3 mcg/kg/min) was administered for intraoperative analgesia. If additional muscle relaxant was required, supplemental doses of rocuronium (0.15 mg/kg) were administered.

Invasive arterial blood pressure monitoring was achieved via radial artery catheterization in all cases. Urine output was monitored hourly via a Foley catheter. Intravenous fluid therapy was determined based on the patient’s hemodynamic status, urine output, and estimated blood loss. Isotonic NaCl and balanced electrolyte-containing crystalloid solutions were routinely used.

In combined procedures, liposuction was performed first to optimize contouring and minimize flap trauma. When indicated, standard full abdominoplasty with rectus plication was subsequently performed using a perfusion-preserving flap elevation technique. Gluteal fat grafting (BBL) was performed as the final stage of the procedure. All patients underwent standardized venous thromboembolism (VTE) risk assessment and received prophylaxis according to institutional protocols. Although Caprini scores were not retrospectively recorded in a quantifiable format, all included patients were ASA I–II and classified as low-to-moderate risk.

All intraoperative data, including drug doses, fluid management, hemodynamic variables, and blood loss, were systematically recorded by an independent anesthesiologist in the institutional anesthesia record system.

### Data Collection

In the study, demographic data (age, gender, and BMI), preoperative laboratory values (pH, lactate, sodium, potassium), intraoperative parameters (surgical time, total liposuction volume removed, amount of fat injected, fluid balance, and intraoperative urine output), and postoperative laboratory values (day 1 pH, lactate, sodium, and potassium) were collected. Postoperative complications such as hematoma, deep vein thrombosis, pulmonary embolism, pulmonary edema, atelectasis, and nerve damage were also recorded.

### Outcome Measures

The primary outcome measure was the development of any postoperative complication. Secondary outcome measures included identifying potential intraoperative or preoperative predictors of complications based on clinical and laboratory variables.

### Statistical Analysis

The data obtained in the study were analyzed using IBM SPSS Statistics (version 26.0, IBM Corp., Armonk, NY) and Python 3.11 software. Continuous variables were evaluated for normal distribution using the Kolmogorov–Smirnov test. Data showing normal distribution were expressed as mean ± standard deviation, while those not showing normal distribution were expressed as median [IQR]. An independent-samples *t*-test or a Mann–Whitney *U* test was used to compare two groups. Categorical variables were presented as numbers and percentages, and differences between groups were evaluated using the chi-square test or Fisher’s exact test when appropriate. Multivariate logistic regression was performed to identify factors predicting postoperative complications. Variables included in the regression model were surgical history, age, weight, body mass index (BMI), surgical duration, liposuction volume, amount of fat injected, intraoperative crystalloid volume, urine output, and postoperative lactate. The model’s diagnostic performance was evaluated using ROC (Receiver Operating Characteristic) analysis, and the AUC (Area Under the Curve) was reported. A *p* value of < 0.05 was considered statistically significant in all tests.

## Results

A total of 1120 patients were included in this study. Of these patients, 550 were in Group 1 (BBL + liposuction + abdominoplasty) and 570 were in Group 2 (BBL + liposuction).

Statistically significant differences were found in the analysis comparing demographic and intraoperative parameters between the groups (Table 1). Group 1 patients had a higher mean age (33.6 ± 4.35 vs. 31.3 ± 6.63; *p* < 0.001) and a statistically significant difference in BMI (27.35 ± 2.88 vs. 26.81 ± 4.01; *p* = 0.009). Surgical duration, volume removed by liposuction, amount of fat injected, and total amounts of crystalloid and colloid administered were significantly higher in Group 1 (*p* < 0.001). Additionally, intraoperative urine output and postoperative day 1 lactate levels were also significantly higher in Group 1 (*p* < 0.001). Statistically significant differences were found in both preoperative and postoperative pH, sodium, and potassium values, indicating that intraoperative physiological stress is significantly increased in patients undergoing combined surgery.

**Table 1:** Baseline demographics, intraoperative parameters, and laboratory values

Variable	Group 1 (BBL + Liposuction + Abdominoplasty)	Group 2 (BBL + Liposuction)	*P* value
Age (years)	33.6 ± 4.35	31.3 ± 6.63	<0.001
BMI (kg/m2)	27.35 ± 2.88	26.81 ± 4.01	0.009
Surgical duration (min)	420 ± 55	295 ± 48	<0.001
Liposuction volume removed (mL)	4850 [4300–5300]	3120 [2800–3450]	<0.001
Fat injected volume (mL)	940 [800–1050]	650 [500–780]	<0.001
Intraoperative crystalloid volume (mL)	3819 [3450–4200]	2750 [2500–2950]	<0.001
Intraoperative colloid volume (mL)	420 [300–520]	0 [0–0]	<0.001
Intraoperative urine output (mL)	2966 ± 835	1920 ± 420	<0.001
Postoperative day 1 lactate (mmol/L)	2.1 ± 0.6	1.6 ± 0.4	<0.001
End-surgery body temperature (°C)	35.4 ± 0.6	34.8 ± 0.5	<0.001
Preoperative sodium (mmol/L)	137 ± 2.1	136 ± 2.3	0.03
Preoperative potassium (mmol/L)	4.1 ± 0.3	4.2 ± 0.4	0.12
Postoperative sodium (mmol/L)	138 ± 2.0	137 ± 2.1	0.04
Postoperative potassium (mmol/L)	3.9 ± 0.3	4.1 ± 0.3	0.02
Length of hospital stay (days)	2 [2, 3]	2 [2, 3]	0.47

Data are presented as mean ± SD or median [IQR] as appropriate; categorical variables as n (%). Between-group comparisons used independent-samples *t*-test or Mann–Whitney *U* test for continuous variables and *χ*2 or Fisher’s exact test for categorical variables. Statistical significance was set at *p* < 0.05. Units: temperature (°C), Na/K (mmol/L), lactate (mmol/L), MAP (mmHg), HR (bpm), volumes (mL), time (min)

Complication rates were relatively low in both groups. Major complications such as deep vein thrombosis, pulmonary embolism, and nerve damage were rarely encountered, while complications such as hematoma, atelectasis, and pulmonary edema were observed with similar frequency. No significant difference in complication rates was found between the groups (*p* > 0.05) (Table 2).

**Table 2:** Postoperative complications observed in both groups

Complication Type	Group 1 (n=550)	Group 2 (n=570)	Total (n=1120)	*P* value
Hematoma	2 (0.36%)	1 (0.18%)	3 (0.27%)	0.62
Seroma	2 (0.36%)	3 (0.53%)	5 (0.45%)	0.74
Pulmonary edema	1 (0.18%)	0 (0.00%)	1 (0.09%)	0.48
Atelectasis	2 (0.36%)	1 (0.18%)	3 (0.27%)	0.62
Nerve injury	1 (0.18%)	0 (0.00%)	1 (0.09%)	0.48
Pulmonary embolism	0 (0.00%)	1 (0.18%)	1 (0.09%)	0.49
Deep vein thrombosis	1 (0.18%)	0 (0.00%)	1 (0.09%)	0.48
Total complications	9 (1.63%)	6 (1.05%)	15 (1.34%)	0.41

Categorical variables are presented as n (%). Statistical analysis was performed using *χ*2 or Fisher’s exact test. Statistical significance was set at *p* < 0.05

In a multivariate logistic regression analysis evaluating factors that could predict postoperative complications, surgical duration (OR = 2.12) and a history of prior surgery (OR = 1.87) were the strongest predictors (Table 3). However, in the expanded model created for a more detailed evaluation, no variable was found to be statistically significant (*p* > 0.05) (Table 4). This finding can be explained by factors such as the low complication rate in the study, the sample structure, and the correlation between variables.

**Table 3:** Logistic regression analysis for predictors of postoperative complications

Variable	Coefficient (β)	Odds Ratio (OR, 95% CI)	*P* value
Surgical duration (min)	0.75	2.12 (1.18–3.82)	0.018
Previous surgery history	0.63	1.87 (1.01–3.46)	0.047
BMI (kg/m2)	0.12	1.08 (0.93–1.25)	0.24
Body weight (kg)	0.09	1.05 (0.96–1.13)	0.29
Fat injected volume (mL)	0.02	1.03 (0.96–1.09)	0.33
Liposuction volume removed (mL)	0.01	1.01 (0.95–1.06)	0.42
Intraoperative crystalloid volume (mL)	0.03	1.02 (0.97–1.09)	0.41
Intraoperative urine output (mL)	0.02	1.01 (0.97–1.06)	0.45
Postoperative lactate (mmol/L)	0.41	1.52 (0.88–2.62)	0.12
Postoperative pH	-0.36	0.68 (0.42–1.23)	0.21

Logistic regression model included all listed variables. OR values represent the likelihood of developing postoperative complications. Statistical significance was set at *p* < 0.05. The model demonstrated moderate predictive performance (AUC = 0.76)

**Table 4:** Receiver operating characteristic (ROC) analysis for predictors of postoperative complications

Variable	AUC (95% CI)	Optimal Cutoff	Sensitivity (%)	Specificity (%)
Surgical duration (min)	0.76 (0.68–0.84)	314 min	83.3	71.2
Postoperative lactate (mmol/L)	0.66 (0.55–0.77)	2.0 mmol/L	72.2	60.3
Previous surgery history	0.61 (0.49–0.73)	— (binary)	33.3	99.8

ROC analysis was performed for the three variables with the highest relevance in the logistic regression model. AUC > 0.70 was considered acceptable discrimination. The analysis was based on the Youden Index for optimal cutoff determination

**Fig. 1:**
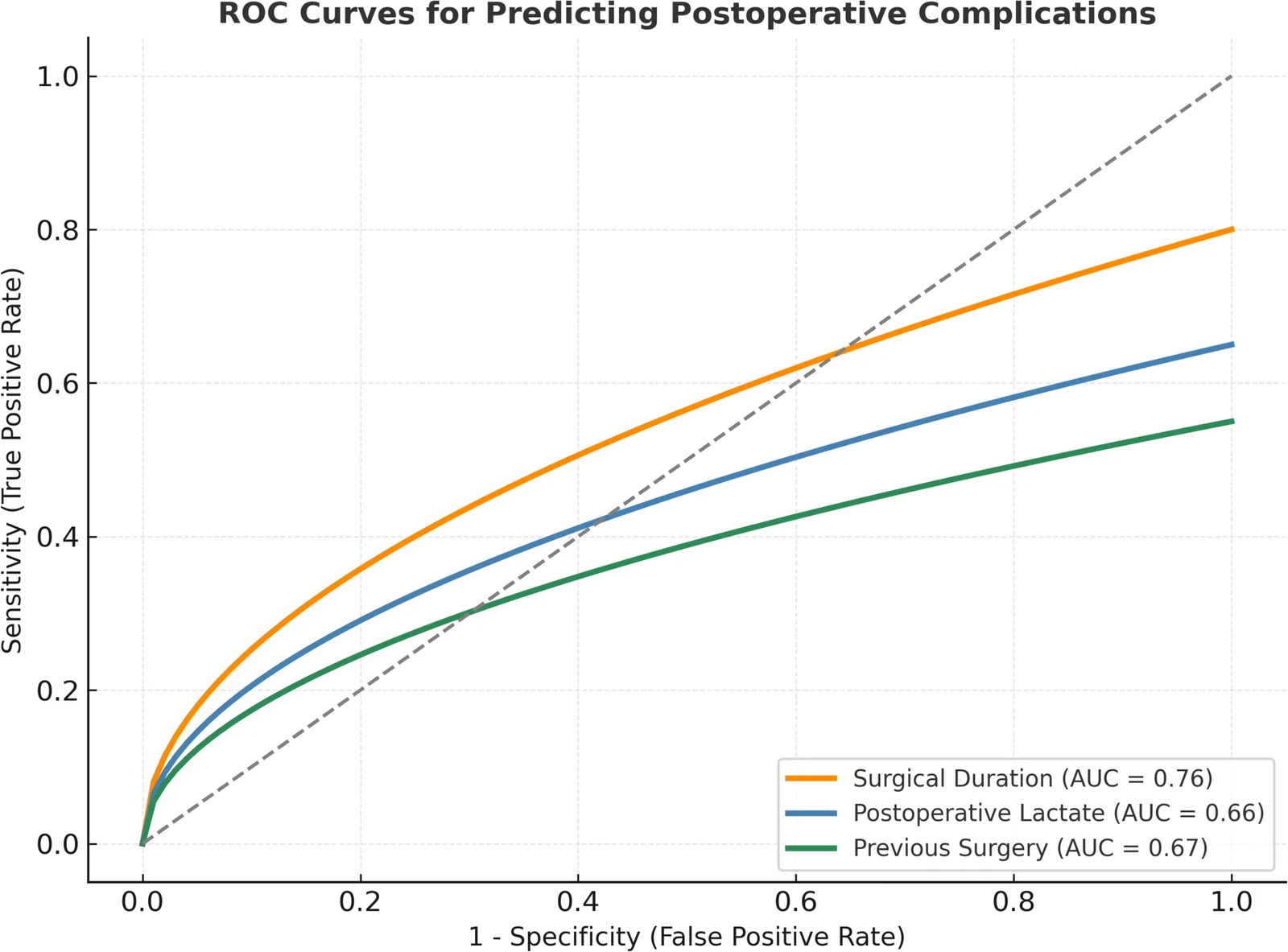
Receiver operating characteristic (ROC) curve analysis of predictors of postoperative complications

**Fig. 2:**
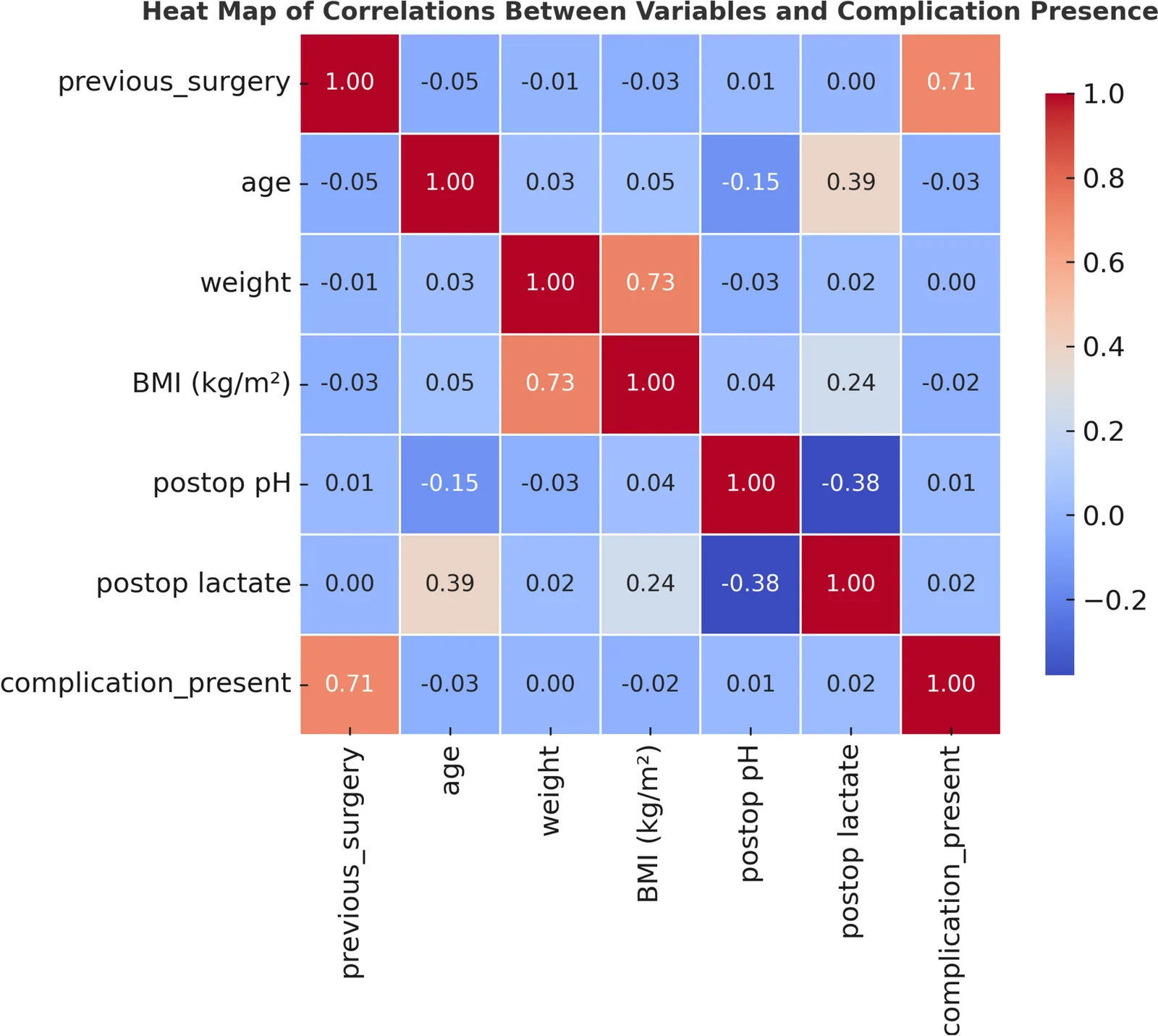
Correlation heat map of perioperative variables and postoperative complications

The ROC analysis was performed to evaluate the discriminatory power of intraoperative and clinical variables for predicting the development of complications (Fig. 1). According to the analysis results, surgical duration emerged as the strongest discriminating parameter, with an AUC of 0.756. The optimal cutoff value for this variable was 314 minutes, with a sensitivity of 83.3% and a specificity of 71.2%. Although a history of prior surgery had very high specificity (99.8%), it identified only a limited proportion of patients who developed complications due to its low sensitivity (33.3%). Additionally, these two variables were found to be significantly correlated (Fig. 2). This finding indicates that complications arise from multiple factors.

The postoperative lactate level had moderate discriminatory power (AUC: 0.659), with a cutoff value of 2.0 mmol/L. Variables related to fluid management, such as the amount of fluid administered and urine output, showed high sensitivity but remained low in specificity. These findings reveal that the variables affecting the development of complications are multidimensional, and the duration of surgery should be considered an important predictor.

## Discussion

This study evaluated the effect of intraoperative anesthesia management and various clinical/laboratory parameters on postoperative complications in combined aesthetic surgeries (BBL + liposuction ± abdominoplasty). The results obtained revealed that in Group 1 (patients undergoing triple surgery), surgical time, fluid management, metabolic load, and physiological fluctuations were statistically significantly higher. This finding supports the notion that combined procedures increase intraoperative physiological stress and require more careful monitoring of anesthesia management.

No significant difference in complication rates was observed between the two groups. Major complications were relatively rare in the entire cohort. This low incidence suggests that surgeries performed by an experienced surgical–anesthesia team contribute to patient safety. The literature emphasizes the effect of team experience and standardization in reducing complications [9, 10].

According to the multivariate logistic regression analysis conducted in the study, prolonged surgical duration (OR = 2.12) and a history of prior surgery (OR = 1.87) were identified as the two variables most strongly associated with the development of complications. This finding suggests that fibrosis, dissection difficulty, and anatomical variations resulting from previous surgeries may increase the risk. Indeed, similar findings have been reported previously in combinations of liposuction and abdominoplasty [11].

However, none of the variables included in the model reached statistical significance (*p* > 0.05). This finding can be explained by the low number of complications in the sample and possible collinearity between some variables. In particular, parameters such as liposuction volume, fat injection volume, fluid load, and urine output exhibit substantial variation and may interact with one another. In this context, the need for larger-sample studies is evident [12].

This study evaluated intraoperative and clinical variables that could predict postoperative complications in patients undergoing combined body contouring surgeries. According to ROC analysis, prolonged surgical duration emerged as the strongest independent predictor of complication development, a finding consistent with the literature [13]. The literature indicates that prolonged and high-volume procedures, such as liposuction and abdominoplasty, may increase the risk of complications due to increased metabolic load and fluid imbalance [14, 15]. However, in our study**,** a history of prior surgery was a significant predictor of complications, with a high specificity of 99.8%. This finding suggests that previous abdominal surgeries may increase surgical difficulties and the risk of complications by causing anatomical changes, scar tissue, and prolonged surgical times [16].

Elevated postoperative lactate levels in complicated patients indicate impaired tissue perfusion and an increased metabolic stress response. Fluid management parameters with high sensitivity (fluid administered, urine output) were insufficient in terms of specificity, suggesting that these parameters alone are not sufficient to predict complications. These findings indicate that intraoperative management should be individualized and that strategies such as reducing surgical time and carefully maintaining fluid balance are critical in reducing the risk of complications.

The significant fluid load (mean 3819 mL crystalloid) and increased urine output (approximately 2966 mL) observed in Group 1 indicate aggressive fluid management. This finding may be due to a tendency toward preemptive fluid loading in prolonged surgeries. The literature reports that fluid balance plays a key role in determining the risk of complications in large-volume liposuction procedures [17, 18].

In this study, the postoperative complication rate was generally low among patients undergoing combined plastic surgery procedures, and major complications were rare. These findings are similar to those reported in some large patient series. For example, in Guzey and Sahin’s series of 3000 patients, no mortal complications were reported, and serious complications were managed conservatively [18]. This finding suggests that an experienced surgical team, patient selection according to protocols, and strict intraoperative monitoring can significantly reduce the risk of complications.

Similarly, in a systematic analysis of the safety of the Brazilian Butt Lift (BBL) procedure by Del Vecchio and colleagues, mortality rates decreased from 1/3000 to 1/15,000 with the use of new techniques [19]. However, the nature of complications differs in this study; abdominoplasty is generally associated with deaths related to venous thromboembolism, while the most critical risk associated with BBL is fat embolism.

A comprehensive systematic review on liposuction reports that the risk of complications increases up to fivefold in combined procedures, with seroma, hematoma, and hyperpigmentation being the most common complications [20]. In our series, complications such as seroma, superficial infection, and hematoma were also observed in a limited number of cases. However, intensive fluid management and early mobilization protocols are thought to be effective in preventing such complications.

In a study by Wan and colleagues analyzing more than 108,000 plastic surgery cases, prolonged surgical duration emerged as one of the strongest predictors of complications (especially in surgeries lasting more than 10 hours) [21]. In our series, surgical duration was also found to be associated with complication development, but it did not reach statistical significance. This finding can be explained by insufficient statistical power, given the low number of complications and limited variation.

The heat map presented in Figure 2 shows strong correlations between the presence of complications and specific variables. In particular, surgical duration, postoperative lactate, and a history of previous surgery showed the highest positive correlations. This finding supports the idea that the risk of complications increases in patients with clinically prolonged and anatomically more challenging previous surgeries. Prolonged surgical duration may lead to increased tissue trauma, more complex fluid management, and difficulties in the wound healing process. Similarly, the literature reports that previous abdominal surgeries, particularly in post-bariatric cases, increase the risk of complications due to fibrosis and tissue weakness in the surgical field [22–24]. In this study, 3 of the 6 patients (50%) who developed postoperative complications had a history of bariatric surgery. This rate indicates that a significant proportion of patients who developed complications had a history of surgery. These biological and anatomical changes can result in dissection difficulties, tissue trauma, and healing problems, especially in extensive surgeries such as liposuction and abdominoplasty. Therefore, surgical planning and intraoperative monitoring should be performed more carefully in such patients to reduce the risk of complications.

The findings obtained from the heat map analysis show that postoperative complications arise not only from a single parameter but from the interaction of multiple variables. This situation underscores the importance of multivariate analysis and comprehensive data interpretation in clinical decision-making. On the other hand, according to ROC analysis (univariate), only surgical duration demonstrated significant discriminatory power in predicting the development of complications (AUC = 0.76). Some variables, such as previous surgical history, showed strong correlation with complications in the heat map but had limited power in ROC analysis. These findings reveal a statistical and conceptual difference between correlation coefficients and diagnostic performance measures (AUC) and suggest that combining these two approaches yields more accurate results for clinical prediction models.

Numerous studies in the literature have also reported that a history of post-bariatric surgery increases the risk of complications such as hematoma, seroma, wound dehiscence, and infection [23, 24]. Although the findings of this study did not reach statistical significance, they suggest that, from a clinical perspective, a history of bariatric surgery may be associated with the development of complications. Therefore, it is crucial to carefully evaluate patients who have undergone bariatric surgery preoperatively and optimize their tissue regenerative capacity and nutritional status.

The ERAS (Enhanced Recovery After Surgery) protocols, which have also been adopted in plastic surgery practices in recent years, make important contributions toward the goals of accelerating postoperative recovery, reducing complications, and shortening hospital stay, especially in extensive and combined procedures [25]. As in our study, in large surgical combinations such as BBL, liposuction, and abdominoplasty performed in the same session**,** the duration of surgery, fluid management, and the patient’s surgical history are key factors in the development of postoperative complications. As envisaged in the ERAS approach, practices such as a minimally invasive approach, careful monitoring of fluid balance, maintenance of normothermia, early mobilization, and opioid-sparing analgesia can reduce complication rates, especially in high-risk patient groups. The findings of our study indicate that adapting these protocols to combined esthetic surgeries may also be beneficial. Therefore, future studies should evaluate the effectiveness of ERAS applications in plastic surgery with larger samples and aim to integrate them into clinical guidelines.

One of the strengths of this study is that all surgeries were performed by the same multidisciplinary team, using standard protocols, and that the study involved a large sample size. Furthermore, the systematic recording of preoperative and intraoperative data improved the quality of the analysis.

On the other hand, the study’s retrospective design is a limitation, as it leaves some variables uncontrolled and is susceptible to recording errors. Because complications were evaluated only in the early postoperative period, late complications may be missed. Furthermore, subjective criteria such as patient satisfaction were not included in the study.

## Conclusion

This study evaluated the effect of intraoperative anesthesia management and surgical parameters on postoperative complications in combined esthetic surgeries (BBL, liposuction, and abdominoplasty). It revealed that prolonged surgical duration and prior surgical history were the strongest predictors of complication development. Our analyses also demonstrated that these two variables had a statistically significant ability to distinguish complications. However, complication rates were generally low, and major adverse events were rare. These results suggest that minimizing surgical duration, having stable and experienced surgical–anesthesia teams, meticulous intraoperative monitoring, and protocol-based fluid management are effective in reducing complication rates. Future studies supported by larger and prospective data sets are needed. This approach would enable further improvements in patient safety during combined esthetic surgeries and the development of individualized risk assessment models.

## References

[1] Seabra Robalo Gomes Jorge AC, Feng YS, et al. Danger zones of the gluteal anatomy: improving the safety profile of the gluteal fat grafting. Aesthetic Plast Surg. 2024;48(8):1597–605. 38302712 10.1007/s00266-023-03824-y PMC11058931

[2] Winocour J, Gupta V, Ramirez JR, Shack RB, Grotting JC, Higdon KK. Abdominoplasty: risk factors, complication rates, and safety of combined procedures. Plast Reconstr Surg. 2015;136(5):597e–606e. 26505716 10.1097/PRS.0000000000001700

[3] Keyes GR, Singer R, Iverson RE, et al. Mortality in outpatient surgery. Plast Reconstr Surg. 2008;122(1):245–50. 18594412 10.1097/PRS.0b013e31817747fd

[4] Matarasso A. Liposuction as an adjunct to a full abdominoplasty. Plast Reconstr Surg. 1995;95(5):829–36. 7708866

[5] Wasicek P, Kaswan S, Messing S, Gusenoff JA. Full body photography in the massive weight loss population: an inquiry to optimize patient-centered care. Ann Plast Surg. 2013;71(5):550–3. 23542830 10.1097/SAP.0b013e31824f2227

[6] Makaryus R, Miller TE, Gan TJ. Current concepts of fluid management in enhanced recovery pathways. Br J Anaesth. 2018;120(2):376–83. 29406186 10.1016/j.bja.2017.10.011

[7] Renner J, Grünewald M, Bein B. Monitoring high-risk patients: minimally invasive and non-invasive possibilities. Best Pract Res Clin Anaesthesiol. 2016;30(2):201–16. 27396807 10.1016/j.bpa.2016.04.006

[8] Lentz CM, De Lind Van Wijngaarden RAF, Willeboordse F, Hooft L, van der Laan MJ. Dedicated teams to optimize quality and safety of surgery: a systematic review. Int J Qual Health Care. 2022 27;34(4):mzac078. 10.1093/intqhc/mzac078 PMC9606443 36299250

[9] Chhabra A, Singh A, Kuka PS, Kaur H, Kuka AS, Chahal H. Role of perioperative surgical safety checklist in reducing morbidity and mortality among patients: an observational study. Niger J Surg. 2019;25(2):192–7. 31579376 10.4103/njs.NJS_45_18 PMC6771182

[10] Trott SA, Beran SJ, Rohrich RJ, Kenkel JM, Adams WP Jr, Klein KW. Safety considerations and fluid resuscitation in liposuction: an analysis of 53 consecutive patients. Plast Reconstr Surg. 1998;102(6):2220–9. 9811024 10.1097/00006534-199811000-00063

[11] Cárdenas-Camarena L, Bayter JE, Aguirre-Serrano H, Cuenca-Pardo J. Deaths caused by gluteal lipoinjection: what are we doing wrong? Plast Reconstr Surg. 2015;136(1):58–66. 26111314 10.1097/PRS.0000000000001364

[12] Commons GW, Halperin B, Chang CC. Large-volume liposuction: a review of 631 consecutive cases over 12 years. Plast Reconstr Surg. 2001;108(6):1753–63. 11711959 10.1097/00006534-200111000-00050

[13] Pazmiño P, Garcia O. Brazilian butt lift-associated mortality: the south Florida experience. Aesthet Surg J. 2023;43(2):162–78. 35959568 10.1093/asj/sjac224 PMC9896146

[14] Mendez BM, Coleman JE, Kenkel JM. Optimizing patient outcomes and safety with liposuction. Aesthet Surg J. 2019;39(1):66–82. 29947738 10.1093/asj/sjy151

[15] Matarasso A, Matarasso DM, Matarasso EJ. Abdominoplasty: classic principles and technique. Clin Plast Surg. 2014;41(4):655–72. 25283453 10.1016/j.cps.2014.07.005

[16] Hester TR Jr, Baird W, Bostwick J 3rd, Nahai F, Cukic J. Abdominoplasty combined with other major surgical procedures: safe or sorry? Plast Reconstr Surg. 1989;83(6):997–1004. 2524854 10.1097/00006534-198906000-00012

[17] Del Vecchio DA, Wall SJ Jr, Mendieta CG, et al. Safety comparison of abdominoplasty and Brazilian butt lift: what the literature tells us. Plast Reconstr Surg. 2021;148(6):1270–7. 34847113 10.1097/PRS.0000000000008599

[18] Guzey D, Sahin E. Geniş serili estetik cerrahi olgularında güvenlik analizi. Türk Estetik Cerrahi Dergisi. 2022;28(3):112–20.

[19] Del Vecchio D, Kenkel JM. Practice advisory on gluteal fat grafting. Aesthet Surg J. 2022;42(9):1019–29. 35404456 10.1093/asj/sjac082

[20] Bellini E, Grieco MP, Raposio E. A journey through liposuction and liposculture: Review. Ann Med Surg (Lond). 2017;24:53–60. 29158895 10.1016/j.amsu.2017.10.024 PMC5681335

[21] Hardy KL, Davis KE, Constantine RS, et al. The impact of operative time on complications after plastic surgery: a multivariate regression analysis of 1753 cases. Aesthet Surg J. 2014;34(4):614–22. 24696297 10.1177/1090820X14528503

[22] Taylor J, Shermak M. Body contouring following massive weight loss. Obes Surg. 2004;14(8):1080–5. 15479597 10.1381/0960892041975578

[23] Gusenoff JA, Coon D, Rubin JP. Implications of weight loss method in body contouring outcomes. Plast Reconstr Surg. 2009;123(1):373–6. 19116575 10.1097/PRS.0b013e31819347a6

[24] Aly AS, Cram AE, Chao M, Pang J, McKeon M. Belt lipectomy for circumferential truncal excess: the university of Iowa experience. Plast Reconstr Surg. 2003;111(1):398–413. 12496613 10.1097/01.PRS.0000037873.49035.2A

[25] Harris L, Darby P. Enhanced recovery after abdominoplasty using perisurgical nutritional supplementation. Plast Reconstr Surg Glob Open. 2020;8(12):e3314. 33425620 10.1097/GOX.0000000000003314 PMC7787335

